# Association between positive history of essential tremor and disease progression in patients with Parkinson's disease

**DOI:** 10.1038/s41598-020-78794-1

**Published:** 2020-12-10

**Authors:** Ruwei Ou, Qianqian Wei, Yanbing Hou, Lingyu Zhang, Kuncheng Liu, Junyu Lin, Zheng Jiang, Wei Song, Bei Cao, Huifang Shang

**Affiliations:** Department of Neurology, Laboratory of Neurodegenerative Disorders, National Clinical Research Center for Geriatrics, West China Hospital, Sichuan University, Chengdu, 610041 Sichuan China

**Keywords:** Translational research, Neurological disorders, Risk factors

## Abstract

This study aimed to explore the effect of pre-existing essential tremor (ET) history on the disease progression of Parkinson’s disease (PD). We recruited and followed-up a group of PD patients from March 2009 to July 2020. The ET history of each patient was obtained by retrospective interviews or past medical records. Cox proportional hazards models with inverse probability of treatment weighting (IPTW) were used to estimate the hazard ratio (HR) with 95% confidence intervals (CIs). Of 785 patients who completed the followed-up visits, 61 patients (7.8%) reported a history of pre-existing ET. Cox regression models after IPTW indicated that the positive ET history in patients with PD was protective against time to United PD Rating Scale III 14-point increase (HR = 0.301, 95% CI = 0.134–0.678, *P* = 0.004), time to akinesia and rigidity 8-point increase (HR = 0.417, 95% CI = 0.218–0.796, *P* = 0.008), time to conversion to Hoehn and Yahr stage 3 (HR = 0.356, 95% CI = 0.131–0.969, *P* = 0.043), time to develop dyskinesia (HR = 0.160, 95% CI = 0.037–0.698, *P* = 0.015), and time to Montreal Cognitive Assessment 3-point decrease (HR = 0.389, 95% CI = 0.160–0.946, *P* = 0.037), but had no relationship with time to tremor 4-point increase (HR = 1.638, 95% CI = 0.822–3.266, *P* = 0.161) and time to death (HR = 0.713, 95% CI = 0.219–2.319, *P* = 0.574). Our study indicated that ET history in patients with PD is associated with a benign prognosis with slower motor and non-motor progression.

## Introduction

The relationship between Essential tremor (ET) and Parkinson’s disease (PD) has been controversially debated in recent years. In the past, they were regarded as two different conditions that represent the most common tremor disorders in adults. However, subsequent studies indicated that the presence of ET significantly increased the risk of conversion to PD^1^. The association between the two conditions has been further strengthened by the findings of Lewy body in the brainstem structures of some patients with ET^2^. Therefore, some scholars regarded the coexist of ET and PD in individuals as Essential tremor-Parkinson’s disease (ET-PD) syndrome, which is characterized by patients with a long-lasting history of ET eventually develop PD. It is reported that the prevalence of subsequent PD in patients with ET ranges from 6.1 to 19%^3–5^, and the mean latency between the onset of ET and subsequent conversion to PD was 22.3 ± 16.8 years based on a retrospective study on 35 ET patients who transferred to PD^6^. At present, clinical characteristics of patients with pre-existing ET and subsequent PD have been greatly discussed. It has demonstrated that ET-PD patients had an older age of onset of PD, less severity of PD, and was on a lower dosage of levodopa compared to PD patients without pre-existing ET^6^.

There is evidence that, when compared with PD patients presenting with postural instability and gait difficulty (PIGD) phenotype, those with tremor-dominant (TD) phenotype are associated with a slower progression of the disease^7^. In addition, the occurrence and progression of non-motor symptoms also vary among different motor phenotypes. Compared with PD patients with TD phenotype, patients with PIGD phenotype are linked to a faster rate of cognitive decline and a higher incidence of dementia^8^. Furthermore, depression and apathy have been reported to be associated with the PIGD phenotype rather than TD phenotype^9^. Patients suffered from ET-PD syndrome usually present with more severe postural and action tremors and can be classified as the PD-TD phenotype^10^. The abovementioned evidences indicate that a history of ET in patients with PD might play a role in modifying the progression of the disease. Therefore, we proposed a hypothesis that a positive history of ET in patients with PD may have a potential impact on the motor and non-motor progression of the disease.

To date, no data has systematically examined the impact of ET history on the progression of PD. Therefore, in the present study, we aimed to recruit and follow up a group of ET-PD patients and a group of PD patients without ET history with a duration of PD < 3 years at baseline to explore the positive ET history on the motor deterioration, onset of dyskinesia, cognitive decline, and survival of PD.

## Results

### Baseline data

A total of 785 patients (407 men and 378 women) were included in the study. Among these patients, 61 (7.8%) reported a history of ET. The mean age of the included patients at enrollment was 60.5 ± 11.8 (61.5 [17.4]) years, with a mean age of PD onset of 58.9 ± 11.8 (60.0 [17.3]) years and a mean PD disease duration of 1.6 ± 0.8 (1.6 [1.4]) years. At baseline, the mean Unified PD Rating Scale (UPDRS) part III score was 24.4 ± 12.2 (24 [16]) and the mean levodopa equivalent daily dosage (LEDD) was 202.1 ± 210.3 (150 [337.5]) mg per day.

### Comparison between patients with and without ET history before and after weighting

Before weighting, the standardized mean differences (SMD) values were greater than 0.1 in Body Mass Index (BMI), sex distribution, age, age of onset of PD, LEDD, levodopa use, dopamine agonist use, UPDRS part III score, motor subtypes, Hoehn and Yahr (H&Y) stage, Frontal Assessment Battery (FAB) score, Montreal Cognitive Assessment (MoCA) score, Hamilton Depression Rating Scale (HDRS) score, and Non-Motor Symptoms Scale (NMSS) score between patients with and without ET history (Table 1).

**Table 1:** Demographic and baseline clinical features between PD patients with and without a positive ET history.

	Survival analysis (n = 785)				Motor and cognitive progression analysis (n = 704)			
	Unweighted sample			Weighted sample	Unweighted sample			Weighted sample
	With ET history (n = 61)	Without ET history (n = 724)	SMD	SMD	With ET history (n = 59)	Without ET history (n = 645)	SMD	SMD
Education	10.2 ± 3.9, 9 (3)	10.0 ± 4.3, 9 (6)	0.056	0.013	10.4 ± 3.7, 10 (4)	10.1 ± 4.2, 9 (6)	0.061	0.006
BMI	22.6 ± 2.6, 22.5 (3.6)	23.1 ± 3.0, 22.9 (3.7)	0.159	0.016	22.7 ± 2.6, 22.5 (3.6)	23.1 ± 2.9, 22.9 (3.8)	0.161	0.017
Sex, male	30 (49.2%)	407 (56.2%)	0.141	0.012	29 (49.2%)	354 (54.9%)	0.114	0.017
Age	58.3 ± 12.1, 57.8 (17.5)	60.7 ± 11.8, 61.8 (17.0)	0.198	0.012	58.0 ± 12.0, 57.8 (17.3)	59.3 ± 11.2, 60.4 (16.8)	0.107	0.012
Age of onset	56.7 ± 12.1, 56.5 (16.6)	59.1 ± 11.8, 60.2 (17.2)	0.198	0.012	56.4 ± 12.0, 56.5 (16.8)	57.7 ± 11.2, 59.1 (16.9)	0.113	0.012
Disease duration	1.6 ± 0.9, 1.3 (1.4)	1.6 ± 0.8, 1.6 (1.4)	0.001	0.001	1.7 ± 0.9, 1.3 (1.4)	1.6 ± 0.8, 1.5 (1.4)	0.091	0.003
LEDD	174.3 ± 208.7, 0 (325)	204.4 ± 210.4, 150 (350)	0.144	0.021	169.4 ± 202.0, 0 (325)	203.9 ± 211.5, 150 (350)	0.167	0.013
Levodopa	27 (44.3%)	378 (52.2%)	0.117	0.021	26 (44.1%)	329 (51.0%)	0.139	0.018
Dopamine agonist	14 (23.0%)	205 (28.3%)	0.160	0.001	13 (22.0%)	190 (29.5%)	0.170	0.009
UPDRS III	21.1 ± 10.9, 22 (17)	24.7 ± 12.3, 24 (17)	0.313	0.026	21.2 ± 11.0, 23 (17)	23.2 ± 11.4, 23 (16)	0.181	0.033
Motor subtypes, TD/Intermediate/PIGD	48/6/7	281/83/360	0.957	0.005	47/6/6	265/75/305	0.937	0.001
H&Y stage	1.8 ± 0.6, 2.0 (0.5)	1.9 ± 0.6, 2.0 (0)	0.166	0.016	1.8 ± 0.6, 2.0 (0.5)	1.9 ± 0.6, 2 (0.5)	0.084	0.022
FAB	16.0 ± 2.0, 16 (2)	15.7 ± 2.4, 16 (3)	0.103	0.003	16.0 ± 1.9, 16 (2)	15.9 ± 2.3, 17 (3)	0.053	0.001
MoCA	24.9 ± 4.0, 26 (4)	24.0 ± 4.4, 25 (6)	0.217	0.021	25.2 ± 3.3, 26 (4)	24.5 ± 4.0, 25 (5)	0.187	0.016
HDRS	8.3 ± 7.5, 6 (10)	9.1 ± 7.8, 7 (10)	0.111	0.016	8.3 ± 7.4, 6 (10)	9.1 ± 7.7, 7 (6)	0.105	0.012
HARS	6.3 ± 5.8, 4 (7)	6.6 ± 5.9, 5 (8)	0.050	0.010	6.3 ± 5.8, 4 (7)	6.6 ± 5.9, 5 (8)	0.048	0.008
NMSS	26.3 ± 28.9, 14 (30)	33.7 ± 28.7, 27 (34)	0.257	0.016	25.4 ± 27.2, 14 (30)	31.8 ± 27.4, 26 (33)	0.235	0.013

PD: Parkinson’s disease. ET: Essential tremor. SMD: standardized mean differences. BMI: body mass index. LEDD: Levodopa Equivalent Daily Doses. UPDRS: Unified Parkinson’s disease Rating Scale. H&Y stage: Hoehn and Yahr stage. FAB: Frontal Assessment Battery. MoCA: Montreal Cognitive Assessment. HDRS: Hamilton Depression Rating Scale. HARS: Hamilton Anxiety Rating Scale. NMSS: Non-Motor Symptoms Scale.

A total of 17 covariates at baseline including education, BMI, sex, age, age of onset of PD, disease duration of PD, LEDD, levodopa use, dopamine agonist use, BMI, motor subtypes, UPDRS III score, H&Y stage, MoCA score, FAB score, HDRS score, Hamilton Anxiety Rating Scale (HARS) score, and NMSS score were included for estimating the propensity score (PS). After weighting, the SMD values of each variable at baseline were reduced. No SMD values were > 0.1, suggesting there was a well between-group balance on baseline characteristics after weighting (Table 1).

### Association of ET history and clinical outcomes

Of the 785 participants who completed the follow-up visits, 94 patients (12.0%) died, with a mean time to death or censoring from baseline of 5.8 ± 2.4 years. For participants with follow-up data on motor assessments or censoring (n = 704), 219 (31.1%) reported an increase of at least 14 points in the UPDRS III score, 81 (11.5%) had tremor 4-point increase, and 274 (38.9%) showed akinesia and rigidity 8-point increase after a mean 3.6 ± 2.2 years of follow-up, while 131 (18.6%) reached H&Y stage ≥ 3 after a mean 5.0 ± 2.3 years of follow up. In addition, 185 patients (26.3%) reported a decrease of at least 3 points in the MoCA score after a mean 3.6 ± 2.2 years of follow-up, and 106 (15.1%) developed dyskinesia after a mean 5.4 ± 2.3 years of follow up. The mean increase scores in the UPDRS-III from the baseline to the follow-up visits were 8.3 ± 10.6 points, while the mean decrease scores in the MoCA were 0.7 ± 3.3 points.

Positive ET history in PD was protective against time to UPDRS III 14-point increase (unweighted *P* = 0.009; weighted *P* = 0.004), time to akinesia and rigidity 8-point increase (unweighted *P* = 0.014; weighted *P* = 0.008), time to conversion to H&Y stage 3 (unweighted *P* = 0.013; weighted *P* = 0.043), time to develop dyskinesia (unweighted *P* = 0.008; weighted *P* = 0.015), and time to MoCA 3-point decrease (unweighted *P* = 0.047; weighted *P* = 0.037), but had no relationship with time to tremor 4-point increase (unweighted *P* = 0.166; weighted *P* = 0.161), and time to death (unweighted *P* = 0.069; weighted *P* = 0.574) (Table 2).

**Table 2:** Univariate Cox models for exploring the association between ET history and clinical outcomes of PD.

Outcomes	Unweighted sample		Weighted sample	
	HR (95% CI)	*P* value	HR (95% CI)	*P* value
Time to death	0.343 (0.109–1.085)	0.069	0.713 (0.219–2.319)	0.574
Time to UPDRS-III 14-point increase	0.340 (0.151–0.766)	0.009*	0.301 (0.134–0.678)	0.004*
Time to tremor 4-point increase	1.703 (0.877–3.305)	0.116	1.638 (0.822–3.266)	0.161
Time to rigidity and akinesia 8-point increase	0.454 (0.241–0.855)	0.014*	0.417 (0.218–0.796)	0.008*
Time to conversion to H&Y stage ≥ 3	0.282 (0.104–0.768)	0.013*	0.356 (0.131–0.969)	0.043*
Time to dyskinesia	0.152 (0.038–0.618)	0.008*	0.160 (0.037–0.698)	0.015*
Time to MoCA 3-point decrease	0.406 (0.167–0.990)	0.047*	0.389 (0.160–0.946)	0.037*

ET: Essential tremor. PD: Parkinson’s disease. UPDRS: Unified Parkinson’s disease Rating Scale. H&Y stage: Hoehn and Yahr stage. MoCA: Montreal Cognitive Assessment.

*Significant difference.

## Discussion

In a sample of patients with early-stage PD, we firstly assessed the association between ET history and disease progression and found that ET history was a protective factor for motor and non-motor progression, especially for akinesia and rigidity deterioration, cognitive decline, and dyskinesia development. However, we found that the presence of ET had no relationship with tremor deterioration and mortality.

Heterogeneity of motor manifestations of PD patients suggested that PD had distinct clinical patterns implying distinct anatomic, biochemical, and pathologic changes^11^. In patients with PD, tremor was often less responsive to dopamine replacement therapy than rigidity and bradykinesia, and had a quite variable response to levodopa^12^. Therefore, tremor was regarded as the motor manifestation which is mostly independent of the other motor signs. Both rest and action tremors can frequently occur in PD, which were associated with multifaceted phenomenology and, possibly, pathophysiology^13^. However, the exact origin of tremor in PD remains unclear. The deficit in the basal ganglia-thalamocortical circuit is one potential tremor generator^14^.

The role of tremor in PD has been disclosed. In patients with PD, tremor is often combined with a family history of parkinsonism, early age of onset, and slower progression^15^. Patients with tremor at onset also had a slower disease progression than those without^7,16^. Furthermore, patients with TD phenotype were not only associated with more severe rest tremor but also had more severe action tremor, suggesting that both rest and action tremor can contribute to the classification of motor phenotype. Therefore, ET-PD patients would be more likely to belong to the TD phenotype of PD^7^. Previous analyses reported that patients with TD phenotype usually had a more benign course compared to those with PIGD phenotype^7^.These discoveries supported our finding that PD patients with a positive history of ET were associated with a slower motor and non-motor progression of the disease. Furthermore, our findings suggested that the role of ET should be considered when defining the PD subtype.

The variable rate of motor sign progression between patients with and without ET history suggested different pathological, genetic, and biochemical mechanisms as well as possible distinct causes for phenotypically different disorders. A pathological link between ET and PD has been established by the finding of Lewy body in a proportion of ET cases^2^. In addition, a 123-I ioflupane SPECT study found evidence of minimal dopaminergic deficits in the caudate nucleus of ET patients^17^, which suggested that some ET patients had a subtle dopaminergic deficit. However, it was still unknown whether ET-PD patients were associated with a slower progression of Lewy body aggregation than PD patients without ET history, which required to be verified by further longitudinal pathologic studies. Moreover, it was also unclear whether genetic factors played a potential role in the disease progression of PD. For example, both Leucine-rich repeat and Ig domain containing 1 gene (*LINGO1*) and its paralog *LINGO2* mutations have been reported to be associated with ET and PD^18^. The significance of hereditary factors on the motor progression of PD remained to be determined.

A new viewpoint suggested that ET-PD syndrome was a distinct clinical entity rather than a combination of the classical phenotype of ET and PD. There was evidence indicating that ET-PD patients had peculiar clinical, neurophysiological, and imaging features^19^. A previous study found that patients with ET-PD had extrapyramidal signs like PD patients with TD subtype, but with more symmetric rest tremor and lower severity of rigidity^19^. Furthermore, the electrophysiological findings in that study^19^ indicated that rest tremor in patients with ET-PD had a synchronous pattern, as in ET with rest tremor, instead of the classical alternating pattern of the rest tremor in PD. If we regarded PD patients with a history of ET as a distinct clinical entity, our findings supported the benign course of this syndrome.

The association between ET history and a slower decline in cognition in our study was consistent with a previous study, which demonstrated that ET was associated with mild deficits in attention, executive functions, memory, and, possibly, other cognitive processes, and most of the clinical series and neurological tests regarded ET as a mildly progressive movement disorder characterized by monosymptomatic tremor^20^. However, the cardinal motor symptoms in PD including tremor, rigidity, bradykinesia, and PIGD often progressed at different rates and showed variable responsiveness to levodopa therapy, challenging the view that baseline motor subtype classification was a significant predictor of death and cognitive decline^21^. The DATATOP study found that there was no difference in performance on neuropsychological tests between patients with TD and PIGD subtypes, suggesting relative independence of motor phenotype and cognitive changes in PD^7^. In addition, a community-based study indicated that patients with TD subtype at baseline did not become demented until they developed PIGD subtype, and dementia did not occur among patients with persistent TD subtype of Parkinsonism^8^. The same study also found that PD patients with PIGD subtype were associated with accelerated cognitive decline and highly increased risk for subsequent dementia, suggesting that PIGD rather than tremor shared common or paralleled neuropathology with dementia^8^.

Some limitations should be recognized. First, some patients (39/61) diagnosed as ET-PD was based on the self-reported history of ET, which might contribute to recall bias. Second, although the robust methods and statistical methods were used, some potential unmeasured confounders might still bias our results. Third, the prevalence of ET history in PD was relatively small and therefore a selection bias cannot be ruled out. Forth, some patients with ET history reported an indefinable onset age of PD, which might contribute to a controversial disease duration of PD. Fifth, the relatively short observation of disease progression of some patients was not sufficient to conclude the influence of ET history on the long-term outcomes.

## Conclusions

Our study indicated that ET history in PD patients was associated with a benign prognosis with slower motor and non-motor progression and had no impact on survival.

## Methods

### Subjects

**Figure 1:**
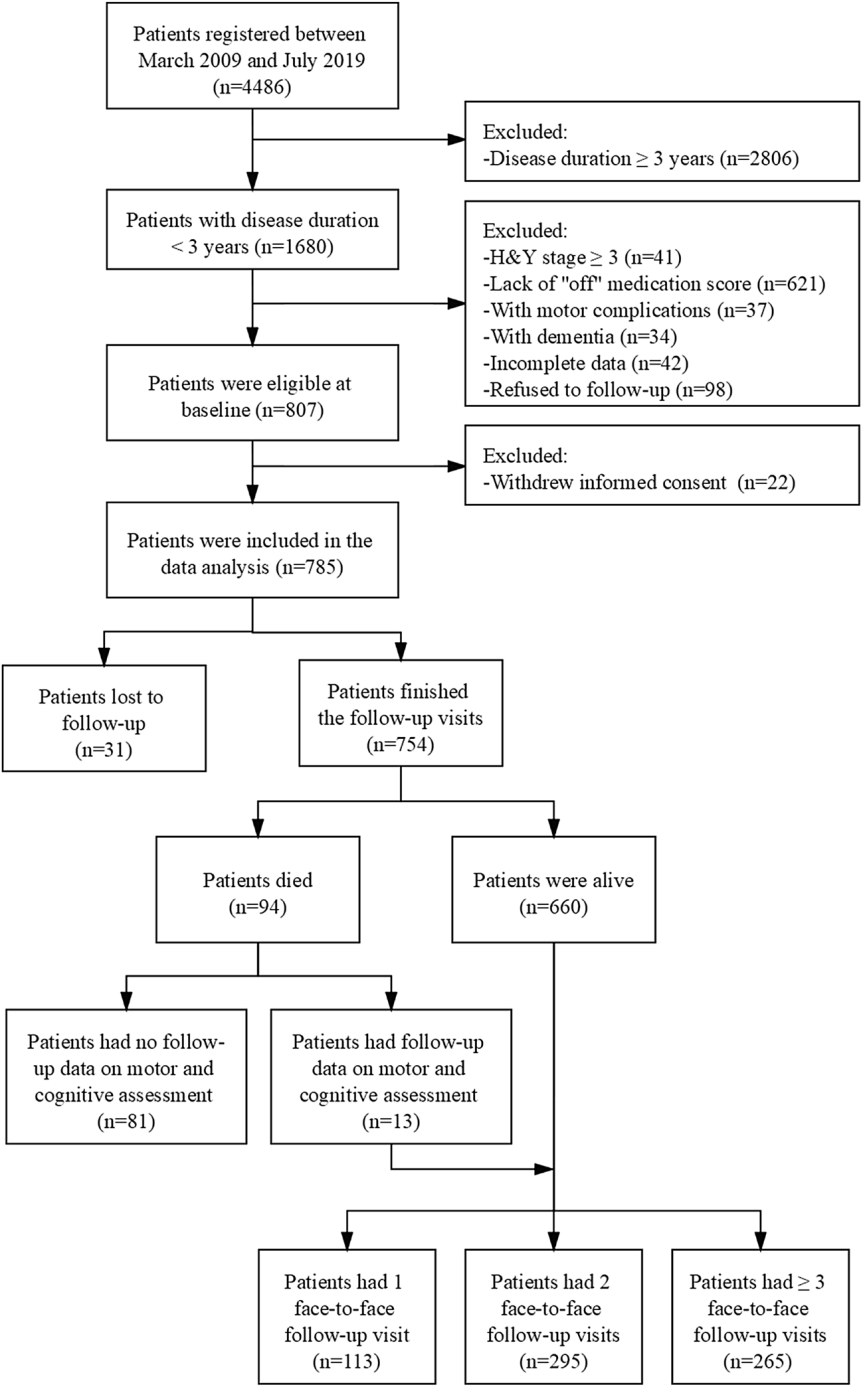
Study flowchart.

All procedures of the study were supported by the Ethics Committee of West China Hospital, Sichuan University (No. 2015236). Initially, 4486 PD patients were seen and registered in the Department of Neurology, West China Hospital of Sichuan University, between March 2009 and July 2019 (Fig. 1). All participants provided written informed consent and met the clinical diagnostic criteria for PD based on the Unified Kingdom PD Society Brain Bank^22^ and also verified by the MDS clinical diagnostic criteria^23^. All the methods were carried out in accordance with the relevant guidelines and regulations.

To explore the influence of ET history on the disease prognosis, participants who met the following inclusion criteria were invited to finish at least one (range 1–10) face-to-face follow-up examination (n = 807): (1) H&Y stage < 3; (2) disease duration < 3 years; (3) assessment at “off” medication; (4) absence of motor complications including motor fluctuation and dyskinesia; (5) absence of dementia. The mean interview intervals for each adjacent visit was set for more than one year (Supplementary Table 1). During the follow-up visit, 22 patients withdraw informed consent, 31 lost contact and 94 died (Fig. 1). Among the 94 patients died, 81 had no face-to-face follow-up data on the motor and cognitive assessments, which was not included as censors in the analysis on the motor and cognitive progression. This is because these patients who had died were probably caused by motor and cognitive progression, which may have a potential effect on the clinical outcomes. Finally, 785 patients who provided information on survival outcomes and 704 patients who had the motor and cognitive outcomes were incorporated into the data analysis.

### Definition of ET-PD

The diagnosis of ET-PD was based on previously defined criteria^24^ and required that: (1) the ET diagnosis was present for > 5 years before the PD diagnosis, (2) the initial ET was characterized by moderate or greater amplitude action tremor without any motor signs of PD, and (3) the initial ET diagnosis occurred in absence of any red flags for possible emerging PD. These subjects with a long-lasting history of ET who developed PD phenotype were classified as ET-PD patients. Past clinical history data on ET history were obtained from patients and their relatives or extracted by their medical records, when available.

### Baseline assessments

At baseline, trained neurologists in our movement disorder center completed a standardized assessment for all patients. Demographic and clinical data including sex, age, height, weight, age of onset of PD, PD duration, years of schooling, and therapeutic schedule were collected. BMI was calculated as body weight (kg) divided by height squared (m^2^). Each patient was classified as TD, Intermediate, or PIGD phenotype based on a previous method^25^. The LEDD for each subject was calculated based on their therapeutic schedule^26^.

The UPDRS part III^27^ and H&Y stage (range 1–5)^28^ were both applied to evaluate the severity of motor symptoms. If possible, all patients were asked to withdraw medications > 12 h at the follow-up visit (484/673, 71.9%). For those patients who did not provide an “off” score, we estimated an “off” medication score by adding the difference value of the study population’s mean “off”-score and mean “on”-score to the patient’s “on” medication score^29^.

The Chinese version of the NMSS^30^ was used to assess the severity of the global NMS burden. In addition, cognitive function was evaluated using the MoCA (range 0–30)^31^ and the FAB (range 0–18)^32^, with lower scores indicating poor cognition. The severity of depression was assessed by the HDRS (24 items)^33^, and the severity of anxiety was evaluated with the HARS^34^.

## Definition of clinical outcomes

### Death

Continuous mortality surveillance was performed mainly throughout the follow-up of patients and their relatives mainly using telephone visits. It lasted until July 1, 2020, which was approximately 11 years after our study began (2009), with as many as 14 years of follow-up for mortality after the patients first diagnosed in 2006. Time to death was defined as the time from the onset of PD to the follow-up visits in which the patient reported the PD-related or non-PD related death.

### Motor decline

It is reported that a change of 2.5–5.2 points on the UPDRS III score is a clinically significant difference^35^, so we defined a fast motor progression as a 14-point increased in the UPDRS-III score (mean of 4-point per year) based on the mean follow-up period from the baseline to the last face-to-face visit or censoring (3.6 ± 2.2 years). Time to the event was defined as the time from the baseline to follow-up visit in which a 14-point increase was first reached. To separately explore the influence of ET history on the tremor and akinesia and rigidity progression of PD, time to tremor 4-point increase and time to akinesia and rigidity 8-point increase were set as another clinical outcomes. The increased score for the tremor or akinesia and rigidity was determined based on the proportion of each symptom in the UPDRS III multiplied by 14. Time to conversion to H&Y stage 3 was set as an additional motor deterioration event, which was defined as the time from the onset of PD to first follow up examinations in which the patient firstly reached a score of H&Y stage 3.

### Dyskinesia

Time to develop dyskinesia was set as the time from the onset of PD to the first followed up examinations in which the patient reported dyskinesia.

### Cognitive decline

Screening for global cognition was performed at each follow-up examination with MoCA. Cognitive decline was defined as a 3-point decrease from the baseline MoCA score and time to the event was defined as the time between baseline and follow-up examinations in which a 3-point decrease was firstly recorded.

### Statistical analyses

Frequencies and descriptive statistics were utilized to summarize the baseline data for the unweighted and weighted samples. Baseline data were reported percentages for categorical variables, and both mean ± standard deviation (SD) and median (quartiles) for continuous variables. The Shapiro–Wilk test was used to check the normality for each variable (Supplementary Table 2).

To balance the differences in various baseline variables between patients with and without ET history, a PS weighting method was selected. The PS model was constructed by conducting a multivariable logistic regression model in which patients with and without ET history was regressed on all baseline variables possibly related to the clinical outcomes, including education, BMI, sex, age, age of onset of PD, disease duration of PD, LEDD, levodopa use, dopamine agonist use, BMI, motor subtypes, UPDRS-III score, H&Y stage, MoCA score, FAB score, HDRS score, HARS score, and NMSS score. The estimated PS was made as to the predicted probability of presenting ET history in each subject. The inverse probability of treatment weighting (IPTW)^23^ was then calculated as the inverse of the PS for the patients with ET history and as the inverse of (1 − PS) for the patients without ET history. This approach created a pseudo-population in which the exposure of ET was independent of measured confounders.

To rate bias reduction after the PS weighting, SMD was compared between patients with and without ET history before and after weighting, with a threshold of < 10% designated to indicate between-group balance. Cox proportional hazards models were used to estimate the HR and examine 95% CIs of the unweighting and weighting samples. The plotted Schoenfeld and time was used to test the proportional hypothesis, and *P* > 0.05 suggested the data met the assumption of equal proportional risk. To visually assess the study groups on the risk of developing a disease milestone, the Kaplan–Meier survival curves were plotted and the log-rank tests were applied (Supplementary Figs. 2–6).

Statistical analyses were programmed by R version 4.0.0 using “Matching”, “survey”, “reshape2”, “survival”, “IPWsurvival”, and “reportReg” packages. All statistical tests were two-tailed, and *P* values < 0.05 were set as statistically significant.

## Supplementary information

Supplementary Information.
