# Supplementary material for: Association between positive history of essential tremor and disease progression in patients with Parkinson's disease
Source: Sci Rep. 2020 Dec 10;10:21749. doi: 10.1038/s41598-020-78794-1 (PMC7729906; doi:10.1038/s41598-020-78794-1)
Supplement: Supplementary file 1 — Supplementary Information. [file 41598_2020_78794_MOESM1_ESM.docx]

**Association between positive history of essential tremor and disease progression in patients with Parkinson's disease**

Ruwei Ou, MD&PhD, Qianqian Wei, MD&PhD, Yanbing Hou, MD&PhD, Lingyu Zhang, MD, Kuncheng Liu, MD, Junyu Lin, MM, Zheng Jiang, MM, Wei Song, MD&PhD, Bei Cao, MD, Huifang Shang^*^, MD

Department of Neurology, Laboratory of Neurodegenerative Disorders, National Clinical Research Center for Geriatrics, West China Hospital, Sichuan University, Chengdu, Sichuan, China

^*^ Correspondence to Huifang Shang, Department of Neurology, Laboratory of Neurodegenerative Disorders, National Clinical Research Center for Geriatrics, West China Hospital, Sichuan University, 610041, Chengdu, Sichuan, China. Email: [hfshang2002@126.com](mailto:hfshang2002@126.com). Telephone: 0086-18980602127. FAX: 0086-028-85423550

**Word count:** main text: 3298; references: 35; abstract: 201

**Running title:** Positive ET history and prognosis of PD

**Keywords:** Parkinson’s disease; Essential tremor; survival; prognosis; motor progression


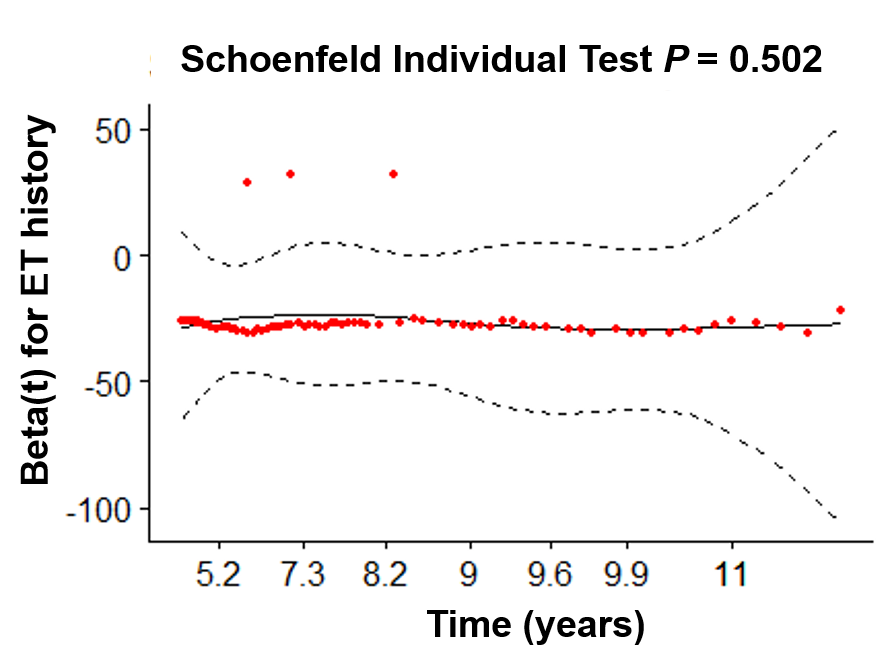


**Supplementary Figure 1 Test for the assumption of equal proportional risk in the survival analysis**

The Schoenfeld individual test indicated that the Cox model that exploring the positive ET history on survival met the assumption of equal proportional risk (*P* = 0.502).


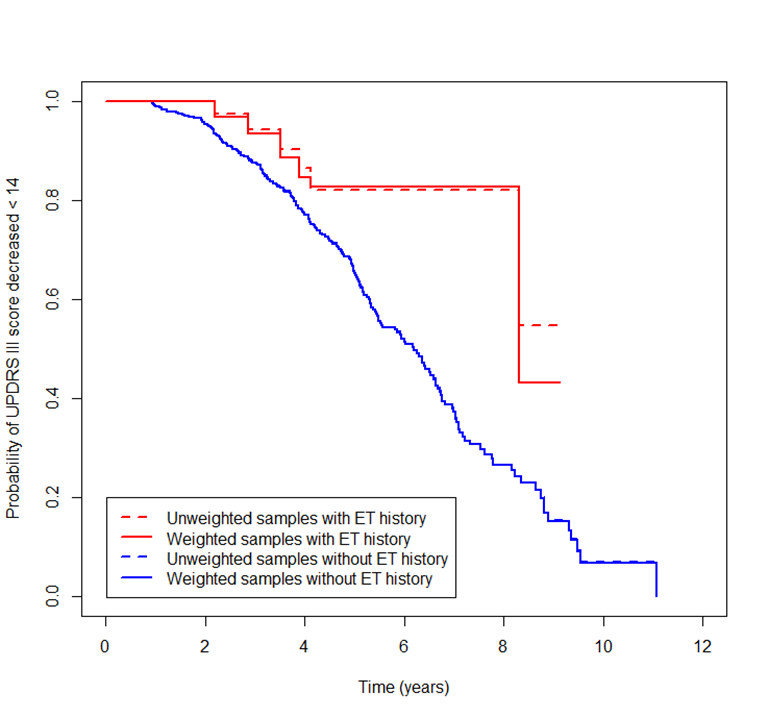


**Supplementary Figure 2 Survival curves for UPDRS III 14-point increase between PD patients with and without a positive ET history before and after weighting**

The Kaplan-Meier curve indicated that patients with a positive ET history had a lower probability of rigidity and akinesia 8-point increase than those without (unweighted *P*=0.006 and weighted *P*=0.012, respectively).

**
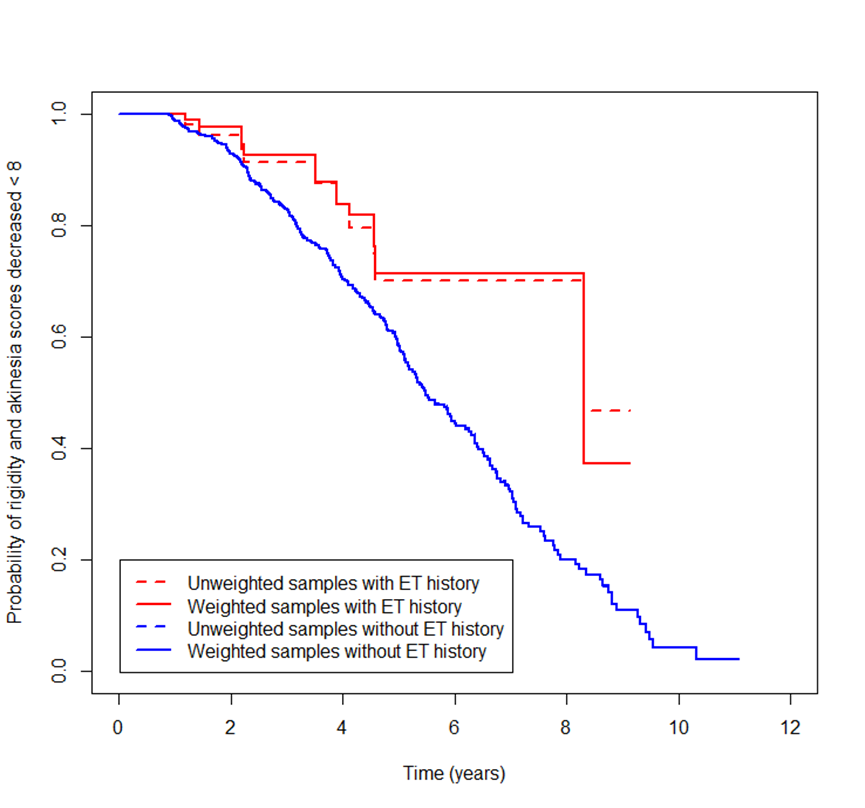
**

**Supplementary Figure 3 Survival curves for rigidity and akinesia 8-point increase between PD patients with and without a positive ET history before and after weighting**

The Kaplan-Meier curve indicated that patients with a positive ET history had a lower probability of rigidity and akinesia 8-point increase than those without (unweighted *P*=0.012 and weighted *P*=0.014, respectively).


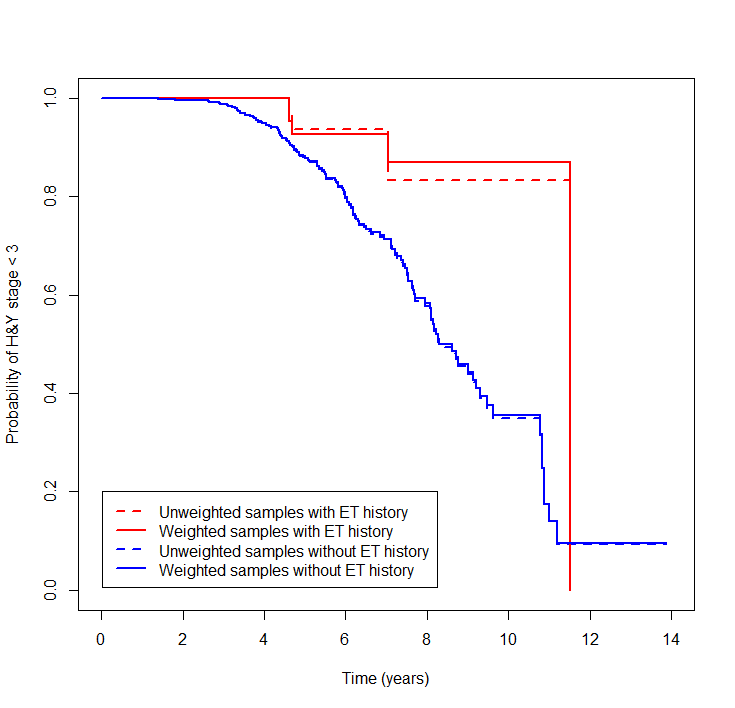


**Supplementary Figure 4 Survival curves for conversion to H&Y stage 3 between PD patients with and without a positive ET history before and after weighting**

The Kaplan-Meier curve indicated that patients with a positive ET history had a lower probability to convert to H&Y stage 3 than those without (unweighted *P*=0.008 and weighted *P*=0.012, respectively).


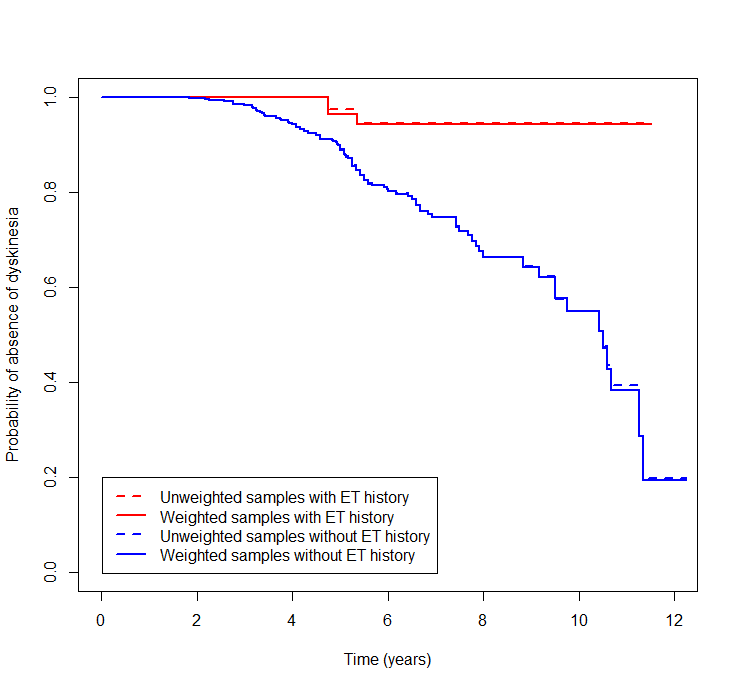


**Supplementary Figure 5 Survival curves for dyskinesia development between PD patients with and without a positive ET history before and after weighting**

The Kaplan-Meier curve indicated that patients with a positive ET history had a lower probability to develop dyskinesia than those without (unweighted *P*=0.002 and weighted *P*=0.004, respectively).


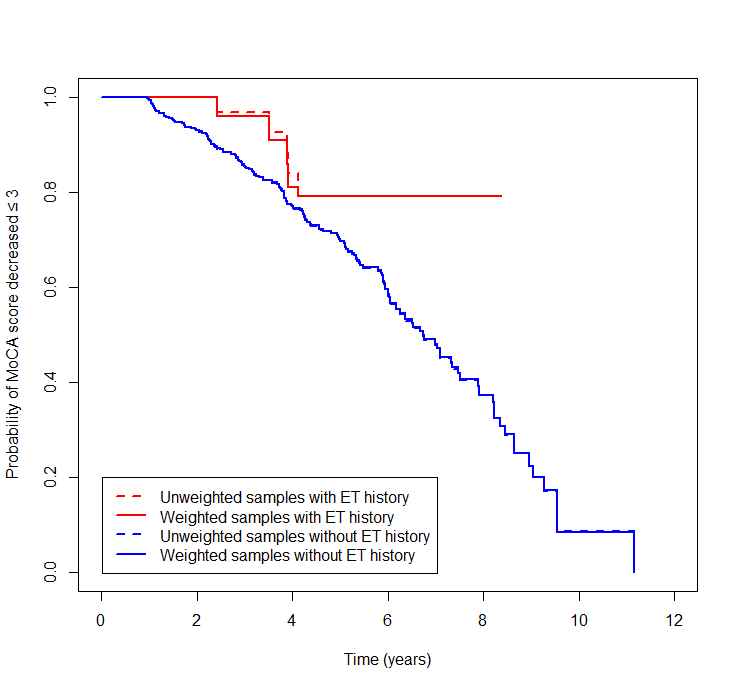


**Supplementary Figure 6 Survival curves for MoCA 3-point decrease between PD patients with and without a positive ET history before and after weighting**

The Kaplan-Meier curve indicated that patients with a positive ET history had a lower probability to decrease 3-point in MoCA than those without (unweighted *P*=0.04 and weighted *P*=0.06, respectively).

**Supplementary Table 1** Interview intervals for each adjacent visit

| Patients had follow-up visits | Number | Interview intervals, years | |
| --- | --- | --- | --- |
|  |  | mean ± SD | median (quartiles) |
| From baseline to follow-up visit 1 | 673 | 1.5 ± 0.9 | 1.6 (0.5) |
| From follow-up visit 1 to 2 | 560 | 1.4 ± 0.6 | 1.5 (0.3) |
| From follow-up visit 2 to 3 | 265 | 1.3 ± 0.3 | 1.4 (0.2) |
| From follow-up visit 3 to 4 | 154 | 1.3 ± 0.2 | 1.3 (0.2) |
| From follow-up visit 4 to 5 | 77 | 1.3 ± 0.2 | 1.3 (0.3) |
| From follow-up visit 5 to 6 | 49 | 1.2 ± 0.2 | 1.3 (0.3) |
| From follow-up visit 6 to 7 | 33 | 1.1 ± 0.2 | 1.1 (0.2) |
| From follow-up visit 7 to 8 | 27 | 1.1 ± 0.1 | 1.1 (0.2) |
| From follow-up visit 8 to 9 | 25 | 1.1 ± 0.1 | 1.1 (0.2) |
| From follow-up visit 9 to 10 | 20 | 1.1 ± 0.1 | 1.1 (0.1) |
| From follow-up visit 10 to 11 | 9 | 1.0 ± 0.1 | 1.0 (0.1) |

SD: standard deviation

**Supplementary Table 2** Shapiro-Wilk normality test for continuous variables at baseline

|  | Data for survival analysis  (n=785) | | Data for motor and cognitive progression analysis (n=704) | |
| --- | --- | --- | --- | --- |
|  | *P-*value | Normal distribution | *P-*value | Normal distribution |
| Education | <0.001 | no | <0.001 | no |
| BMI | 0.184 | yes | 0.069 | yes |
| Age | 0.004 | no | 0.003 | no |
| Age of onset | <0.001 | no | <0.001 | no |
| Disease duration | <0.001 | no | <0.001 | no |
| LEDD | <0.001 | no | <0.001 | no |
| UPDRS III | 0.018 | no | <0.001 | no |
| FAB | <0.001 | no | <0.001 | no |
| MoCA | <0.001 | no | <0.001 | no |
| HDRS | <0.001 | no | <0.001 | no |
| HARS | <0.001 | no | <0.001 | no |
| NMSS | <0.001 | no | <0.001 | no |

BMI: body mass index. LEDD: Levodopa Equivalent Daily Doses. UPDRS: Unified Parkinson’s disease Rating Scale. FAB: Frontal Assessment Battery. MoCA: Montreal Cognitive Assessment. HDRS: Hamilton Depression Rating Scale. HARS: Hamilton Anxiety Rating Scale. NMSS: Non-Motor Symptoms Scale.

*P* > 0.05 indicated the variable met normal distribution.
